# COVID-19 Related Knowledge and Mental Health: Case of Croatia

**DOI:** 10.3389/fpsyg.2020.567368

**Published:** 2020-11-23

**Authors:** Marko Galić, Luka Mustapić, Ana Šimunić, Leon Sić, Sabrina Cipolletta

**Affiliations:** ^1^Department of Psychology, University of Zadar, Zadar, Croatia; ^2^Department of General Psychology, University of Padua, Padua, Italy

**Keywords:** COVID-19, COVID-19 related knowledge, coronavirus, mental health, anxiety, depression, optimism, pessimism

## Abstract

**Background and Aims:** The COVID-19 pandemic has led to radical and unexpected changes in everyday life, and it is plausible that people’s psychophysical health has been affected. This study examined the relationship between COVID-19 related knowledge and mental health in a Croatian sample of participants.

**Methods:**

An online survey was conducted from March 18 until March 23, 2020, and a total of 1244 participant responses were collected (85.5% were women and 58.4% completed secondary education). Measures included eight questions regarding biological features of the virus, symptoms, and prevention, the Hospital Anxiety and Depression Scale, and Optimism-Pessimism Scale. According to the answers given on the questions on COVID-19 related knowledge, participants were divided in two groups: (1) informed and (2) uninformed on each question. They were then compared in the expressed levels of anxiety, depression, pessimism, and optimism. Full vs. partial mediation models with optimism/pessimism as a mediator in the relationship between anxiety/depression and the accuracy of responses for questions about handwashing and ways of transmission were estimated.

**Results:**

Participants who responded correctly on the question about handwashing had higher levels of anxiety, depression, and pessimism than those participants whose answer was incorrect, while participants who answered correctly on the question about the percentage of patients who develop serious breathing problems had higher levels of depression than those who answered incorrectly. Lower levels of anxiety and pessimism were observed in the participants who answered correctly about ways of transmission. Higher levels of pessimism were found in participants who scored incorrectly on questions about the efficiency of antibiotics, most common symptoms, and the possibility of being infected by asymptomatic carriers. Higher levels of knowledge about handwashing were predicted by higher levels of anxiety and pessimism. Higher levels of knowledge about ways of transmission were predicted by lower levels of anxiety and lower levels of pessimism. The examined relationships between anxiety/depression and knowledge were mediated by pessimism.

**Conclusion:**

The findings of this study suggest that knowledge about COVID-19 may be useful to reduce anxiety and depression, but it must be directed to the promotion of health behaviors and to the recognition of fake news.

## Introduction

Since the beginning of 2020, people’s daily lives have fundamentally changed. Everyone is well aware that the cause of such a change was the spread of a novel coronavirus (SARS-CoV-2) that initially appeared in the Chinese city of Wuhan during December 2019 (Politico Magazine, 2020; World Health Organization, 2020a). Since then, the virus has spread all across the world, resulting with a declaration of a pandemic on March 11, 2020, by the World Health Organization (2020b). It is known that being infected by the novel coronavirus causes COVID-19, a respiratory disease that can ultimately lead to fatal outcomes. However, it is not currently possible to estimate the prevalence of the disease with precise certainty, given the fact that in many cases an infected person does not show any symptoms, i.e., for every COVID-19 confirmed case there are multiple undetected ones (Li R. et al., 2020). According to currently available data (John Hopkins University, 2020), mortality rates vary from one area to the other: for example, by September 2020 the mortality rate in Italy was 13.1%, in the United Kingdom 12.2%, in Belgium 11.5%, while in Kuwait the mortality rate was 0.6%, in Bahrain 0.4%, and in Vietnam 0.3% (John Hopkins University, 2020).

In an attempt to deal with the potentially fatal consequences of the pandemic, many countries have decided to implement a variety of strategies that include different forms of economic measures, along with a strong emphasis on social contacts restrictions (Bzdok and Dunbar, 2020). Although human society had been confronted with various forms of infectious diseases from the earliest days, it can be said that it has never before, on such a global level, been faced with restrictions that fundamentally change their everyday lives (Hu et al., 2020). Even though people were expected to avoid public spaces and increase indoor time, there were also favorable life changes (e.g., frequent physical exercise, increased fruit, and vegetable intake) in addition to the unfavorable ones (e.g., increased screen time) (Hu et al., 2020).

In such a situation, the importance of preserving physical health is constantly being stressed, and new challenges such as health care disparities, losing housing, limited access to food, as well as disrupted life plans (Cipolletta and Ortu, 2020; Fraenkel and Cho, 2020) need to be tackled. Having said that, governments are urged to address the impact of the pandemic on mental health (United Nations, 2020).

A large number of studies examined the impact of the lockdown on mental health (Branley-Bell and Talbot, 2020; Cellini et al., 2020; Mechili et al., 2020; Pieh et al., 2020; Verma and Mishra, 2020), as well as its relation with certain constructs such as anxiety and depression. Adams-Prassl et al. (2020) reported negative quarantine effects on the mental health of the United States population while other researchers found that lockdown affects sleep quality (Huang and Zhao, 2020; Rossi et al., 2020) and that higher levels of anxiety can be explained by the time spent reading and discussing news about COVID-19 (Rosen et al., 2020). Previous outbreaks of infectious diseases, such as SARS, have shown a significant potential for psychological contagion, which often lead to widespread fear, anxiety, and a variety of psychological problems (Liu et al., 2020). These problems may include posttraumatic stress disorder (Bo et al., 2020), a decrease in personal interest (Shi et al., 2003), stigmatization (Mak et al., 2010), and an increase in the suicide rate (Cheung et al., 2008).

The previously mentioned SARS epidemic (Leung, 2003) and the more recent Ebola virus epidemic (Ajilore et al., 2017) highlighted the importance of knowledge about the cause and symptoms of the disease for practicing precautionary measures. It is important to emphasize the role of knowledge about the latter and the treatment when studying the effect of the COVID-19 pandemic on mental health. People are exposed to a large amount of both real and fake information on a daily basis, leading to confusion that may create a panic state, which is often a greater danger than the disease risk (Depoux et al., 2020). A study (Gao et al., 2020) on the Chinese population found that those who are frequently exposed to social media are more likely to experience anxiety and depression since they have greater access to information (Qiu et al., 2020). Zhou et al. (2020) emphasize that misinformation and fabricated reports increase depression levels.

The novel coronavirus as well as the situation the world has been encountering since the declaration of the pandemic bring a great uncertainty and fear of the unknown (Cipolletta and Ortu, 2020), which lead to an increase of anxiety levels not only among those with preexisting mental health conditions but also among healthy individuals (Asmundson and Taylor, 2020; Lee et al., 2020; Shigemura et al., 2020). Furthermore, it was shown that the prevalence of negative emotions (e.g., anxiety, depression, and indignation) and sensitivity to social risks increased, while the scores on positive emotions and life satisfaction decreased (Li S. et al., 2020). Wang et al. (2020) reported that more than half of their study participants rated the psychological impact of the outbreak as moderate to severe; one quarter of the respondents reported moderate to severe anxiety symptoms. Except for the aforementioned states caused by the emergence of the novel coronavirus, stereotyping (Lima et al., 2020) and discrimination (Hahad et al., 2020) occurred as well. Some researchers suggest that panic attacks, psychosis, and suicidal thoughts may also be experienced (Salari et al., 2020). According to the literature review by Brooks et al. (2020), a lockdown period requires efficient and rapid communication, which would allow quarantined people to understand the situation by providing them all of the necessary information.

Geldsetzer (2020) reported that the general knowledge of United Kingdom and United States respondents about the novel coronavirus is good, with misconceptions such as the use of antibiotics to stay protected from the infection. Moreover, very good knowledge of Iranian medical students is reported by Taghrir et al. (2020), along with a high percentage of those who practice preventive behaviors (94.47%), which was significantly negatively correlated with the perception of disease risk. Zhong et al. (2020) report high scores among the Chinese population on a COVID-19 knowledge test, while the Indian population showed moderate levels of knowledge about the COVID-19 infection and adequate knowledge about its preventive aspects (Roy et al., 2020). Chockalingam et al. (2020) pointed out that male and female students do not differ in the level of their COVID-19 knowledge, while Banda et al. (2020) found misconceptions about the mode of transmission and disease severity among Malawi respondents. Findings about the association between knowledge and practiced behaviors are controversial. In a study by Brug et al. (2004), there was no significant association between behavior and SARS knowledge, while Lau et al. (2007) reported that hospital avoidance was associated with misconceptions about the mode of transmission. According to Shi et al. (2003), positive and negative information about the infectious disease affect risk perception and behaviors differently: positive information (suggests positive consequences, such as new recovery cases) maintains mental health and rational coping behavior, while negative information (notifications about negative consequences, e.g., number of new cases) increases the risk perception level and leads to irrational fear and nervousness.

Results of previous studies also showed that unrealistic optimism can lead to an underestimation of risk and illness (Makridakis and Moleskis, 2015). Chang and Sivam (2004) reported that defensive pessimism had a direct positive effect on SARS related fears, which were related to immediate preventive health behaviors. Raude et al. (2020) showed that Europeans tend to be overly optimistic about the novel coronavirus, while Zhou et al. (2020) found that optimistic thoughts and attitudes toward the development of the pandemic are a protective factor against anxiety and depression. Jovančević and Milićević (2020) report that higher levels of respect toward measures taken against COVID-19 spreading are predicted by higher levels of optimism. Moreover, Arslan et al. (2020) suggested that higher levels of optimism and lower levels of pessimism may reduce the negative impact of psychological inflexibility on anxiety, depression, and somatization.

The first COVID-19 case in Croatia was registered on February 25. The lockdown started March 19 when the number of registered cases was 105 and 5 people had recovered up to that date. The lockdown, which, according to the University of Oxford (2020), was the strictest in the world among other measures, included the prohibition of all public gatherings with more than five people, and citizens were allowed to leave their city or municipality only for work obligations. The measures started to ease on April 27 when there were 873 infected cases and 1166 recovered cases. A further ease of measures was implemented in the following 2 weeks. To the best of our knowledge, there is no research up to date on the aspects of mental health considering knowledge on different types of information on COVID-19 in Croatia. Moreover, only a few studies (Du et al., 2020; Wang et al., 2020; Yıldırım and Güler, 2020) have explored the relationship between knowledge and mental health during the COVID-19 pandemic, and no study has yet explored the relationship with optimism and pessimism.

The aim of this study was to examine COVID-19 related knowledge and its relationship with anxiety, depression, optimism, and pessimism on a Croatian sample of participants. On the basis of the well-recognized protective effect of knowledge (Wang et al., 2020; Yıldırım and Güler, 2020), we hypothesized that higher levels of anxiety, depression, and pessimism would be related with minor knowledge related to COVID-19. We also expected that participants with higher levels of optimism would be less informed and less anxious and that anxiety would be positively correlated with pessimism. Our ultimate hypothesis was that optimism/pessimism could be viewed as a mediator in the relationship between anxiety/depression and knowledge related to COVID-19.

## Materials and Methods

### Participants and Procedure

Participants were recruited by using the snowball method. The study survey was advertised in different Facebook groups as well as on the WhatsApp messaging application. The total number of participants was 1296. Fifty-two of them were not included in the analysis because they filled out the survey after the date chosen for closing data collection. Of the remaining 1244 respondents, 85.5% were female, 58.4% completed secondary education, and the average age was 36.49 (SD = 12.76). A description of the study sample is shown in Table 1.

**TABLE 1:** Sociodemographic and psychometric characteristics of the population.

	Overall	Anxiety			Depression		
		Normal	Border	Abnormal	Normal	Border	Abnormal
Age	36.49	36.93	35.66	36.21	36.18	36.56	38.88
**Sex**							
Male	180 (14.5%)	136 (75.6%)	31 (17.2%)	13 (7.2%)	149 (82.8%)	24 (13.3%)	7 (3.9%)
Female	1064 (85.5%)	558 (52.5%)	249 (23.4%)	257 (24.2%)	787 (74.0%)	168 (15.8%)	109 (10.2%)
**Education**							
Elementary	20 (1.6%)	4 (20%)	4 (20%)	12 (60%)	11 (55%	3 (14%)	6 (30%)
Secondary	726 (58.4%)	407 (56.1%)	160 (22.0%)	159 (21.9%)	547 (75.3%)	112 (15.4%)	67 (9.2%)
Undergraduate	180 (14.5%)	101 (56.1%)	42 (23.3%)	37 (20.6%)	134 (74.4%)	32 (17.8%)	14 (7.8%)
Graduate	282 (22.7%)	282 (56.9%)	67 (23.8%)	57 (20.2%)	216 (76.6%)	40 (14.2%)	26 (9.2%)
Postgraduate	36 (2.9%)	24 (66.7%)	7 (19.4%)	5 (13.9%)	28 (77.8%)	5 (13.9%)	3 (8.3%)
**Significant life event**							
Yes	303 (24.4%)	133 (43.9%)	80 (26.4%)	90 (29.7%)	205 (67.7%)	61 (20.1%)	37 (12.2%)
No	941 (75.6%)	561 (59.6%)	200 (21.3%)	180 (19.1%)	731 (77.7%)	131 (13.9%)	79 (8.4%)
Infection prevention and control measures	63 (5.1%)						
Quarantine	15 (1.2%)	7 (46.7%)	5 (33.3%)	3 (20%)	13 (86.7%)	1 (6.7%)	1 (6.7%)
Self-imposed isolation	48 (3.9%)	20 (41.7%)	14 (29.2%)	14 (29.2%)	28 (58.3%)	14 (29.2%)	6 (12.5%)
Chronic diseases	126 (10.1%)						
Heart disease	44 (3.5%)	19 (43.2%)	17 (38.6%)	8 (18.2%)	25 (56.8%)	9 (20.5%)	10 (22.7%)
Respiratory disease	51 (4.1%)	25 (49.0%)	10 (19.6%)	16 (31.4%)	35 (68.6%)	7 (13.7%)	9 (17.6%)
Diabetes	21 (1.7%)	8 (38.1%)	6 (28.6%)	7 (33.3%)	15 (71.4%)	1 (4.8%)	5 (23.8%)
More than one	10 (0.8%)						

The data was collected via Google Forms survey from March 18 until March 23, 2020. These dates were chosen because 3 weeks had passed since the first registered case in Croatia, the lockdown had been announced, and nobody had investigated the knowledge about the novel coronavirus among the general population yet. Data collection was originally thought to last for a week, but due to the earthquake in Zagreb (March 22, 2020), which was not included as a significant event among the answers to the question regarding significant life events, researchers decided to stop collecting data. In the week prior to filling out the survey, 303 participants (24.4%) had experienced a significant life event such as changes at work, death of a close person, or breaking up a close relationship. Only 5.1% of participants were under infection prevention and control measures. The study was approved by the Ethical Committee of the Department of Psychology at the University of Zadar. Before starting the survey, participants were informed about the study details. Informed consent was signed by ticking a box at the bottom of the first page in Google Forms, before the beginning of the survey. Participants were able to withdraw their data by contacting the research team via provided e-mail addresses.

### Measures

The authors of the study used the information available on the WHO website to examine knowledge about COVID-19.^1^ Eight questions were used to examine the participants’ knowledge about the coronavirus; five of them were multiple choice questions and three questions were true/false type questions. All the questions (presented in the Supplementary Material) were translated from English to Croatian by using back translation. One point was given for every correct answer and 0 points were given for incorrect answers. The initial plan was to make a linear combination of answers to these eight questions as a total score that would indicate the subject’s knowledge on the coronavirus. Various types of factor and reliability analyses were performed to see whether a linear combination of the results could be used, but the results did not support this. It was then decided to consider each question as separate and to examine the relationship of response accuracy for each question with the research variables. Participants were divided in two groups for each of the eight questions according to their answers. More specifically, if participants scored correctly on question 2 but incorrectly on question 3, they were put in the “informed” group for question 2 and the “uninformed” group for question 3.

The Hospital Anxiety and Depression Scale (HADS; Zigmond and Snaith, 1983) is divided into the Anxiety subscale and the Depression subscale. Both subscales contain seven items. Responses were given on a 4 point Likert scale with the answer 0 meaning *not at all* and 3 meaning *most of the time*. According to Bjelland et al. (2002), this instrument performs well in the general population. In this study, the internal reliability measured by the Cronbach alpha coefficient was 0.88 for the Anxiety subscale and 0.75 for the Depression subscale. The scale was previously validated on a Croatian sample by Pokrajac-Bulian et al. (2015).

The Optimism-Pessimism Scale (OPS) was developed by Penezić (2002) to measure positive and negative expectations of future activities outcome. This scale consists of the Optimism subscale with six items and the Pessimism subscale with eight items. Responses were given on a 5 point Likert scale with the answer 1 meaning *strongly disagree* and 5 meaning *strongly agree*. The internal reliability measured by the Cronbach alpha coefficient in this study was 0.82 for the Optimism subscale and 0.86 for the Pessimism subscale.

### Statistical Analysis

The first step in the data analysis was to check the descriptive statistics of the examined variables and conduct difference tests (the *t*-test and Welch’s *t*-test) and correlational analyses (Pearson, Point-biserial, and Phi coefficients of correlation) using the program STATISTICA 13.5. The *t*-tests and Welch’s *t*-tests were conducted to examine the differences in anxiety, depression, optimism, and pessimism between groups of respondents who provided and did not provide a correct response to a question about COVID-19. Correlational analyses were conducted to examine the relationships between sex, age, educational status, the existence of significant life events, prevention and control measures, and of chronic diseases, anxiety, depression, optimism, pessimism, and the accuracy of the responses to the COVID-19 questions.

Models proposing optimism/pessimism as a mediator in the relationship between anxiety and depression on the one side and response accuracy on the other side were tested. The models and their significance were estimated by conducting path analysis using the program Mplus 6.12 (Muthén and Muthén, 2010), with the WRMR (weighted root mean square residual) method of parameter estimation. WRMR is a badness of fit index, which means that a smaller index value indicates better fit (DiStefano et al., 2017). This method of parameter estimation was used due to the categorical (dichotomous) variable included in the models, that is, the correct or incorrect answer on the given question. Therefore, the path analyses conducted were a combination of linear and probit regression. The accepted statistical significance level for this research was *p* < 0.05 to reject the research’s null hypotheses, in which the researchers only accept 5% of error to reject a null hypothesis.

## Results

The percentage of correct answers to the questions about COVID-19, the means and standard deviations on the scales measuring anxiety, depression, optimism, and pessimism are reported in Table 2. The percentage of correct answers to questions 2, 4, 7, and 8 is higher than 88%, whereas for questions 1, 3, and 5 the percentage of correct answers ranges from 50–69.99% (the questions are reported in the Supplementary Material). The lowest percentage of correct answers was achieved on question 6; only 8.6%, so this question can be considered the most difficult of all. Mean levels of anxiety (*M* = 7.25, SD = 4.25) and depression (*M* = 5.34, SD = 3.63) can be considered as normal, with respect to the criteria of the HADS. The mean levels of optimism and pessimism were 19.86 (SD = 6.93) and 22.91 (SD = 4.33) with the possible range for optimism being 8–40 and 6–30 for pessimism, respectively.

**TABLE 2:** Descriptive statistics of observed variables (*N* = 1244).

	*M*	SD
1. Effects of rinsing nose	0.58	0.50
2. Efficacy of antibiotics in preventing COVID-19	0.98	0.14
3. The most common symptoms of COVID-19	0.70	0.46
4. Handwashing to protect from COVID-19	0.88	0.32
5. COVID-19 ways of transmission	0.52	0.50
6. The percentage of COVID patients that develop serious breathing problem	0.09	0.28
7. Persons without symptoms can transmit COVID	0.97	0.18
8. Virus time of survival on surfaces	0.94	0.24
Anxiety	7.25	4.25
Depression	5.34	3.63
Optimism	19.86	6.93
Pessimism	22.91	4.33

*Means can be observed as proportions of correct answers for questions 1–8.*

According to the answer given on each question about COVID-19, participants were divided in two groups and compared in the expressed levels of anxiety and depression (Table 3) and pessimism and optimism (Table 4). Participants who scored correctly on question 4 (The best way to protect from COVID-19 is to wash hands regularly) had higher levels of anxiety, depression, and pessimism than participants who did not give a correct answer to this question. Participants who gave an incorrect answer on question 5 (COVID-19 is transmitted by) had higher levels of anxiety and pessimism than participants whose answers were correct. On question number 6 (What is the percentage of COVID patients that develop serious breathing problems?), participants with an incorrect answer had lower levels of depression than participants who scored correctly. Participants who scored correctly on questions 2 (Efficacy of antibiotics in preventing COVID-19), 3 (The most common symptoms of COVID-19), and 7 (Persons without symptoms can transmit COVID) had lower levels of pessimism than those who scored incorrectly.

**TABLE 3:** The results of *t*-tests (with Welch’s correction) to examine differences in the observed level of anxiety and depression between those with correct and incorrect answers on each question (*N* = 1244).

	Anxiety				Depression			
	*M*_*True*_	*M*_*False*_	*t*	df	*M*_*True*_	*M*_*False*_	*t*	df
1. Effects of rinsing nose	7.30	7.18	0.48	1176.34	5.31	5.38	−0.33	1183.78
2. Efficacy of antibiotics in preventing COVID-19	7.25	7.12	0.15	25.90	5.36	4.46	1.27	26.12
3. The most common symptoms of COVID-19	7.13	7.51	−1.46	732.32	5.30	5.43	−0.57	669.97
4. Handwashing to protect from COVID-19	7.52	5.21	−7.22**	205.12	5.48	4.33	−5.01**	250.40
5. COVID-19 ways of transmission	6.83	7.70	−3.59**	1228.82	5.19	5.51	−1.57	1214.79
6. The percentage of COVID patients that develop serious breathing problem	7.99	7.18	−1.83	124.93	6.19	5.26	−2.22*	121.12
7. Persons without symptoms can transmit COVID	7.25	7.10	0.22	41.58	5.35	4.98	0.73	42.44
8. Virus time of survival on surfaces	7.27	6.95	0.63	84.97	5.38	4.76	1.56	87.26

*p < 0.05, **p < 0.01.

**TABLE 4:** The results of *t*-tests (with Welch’s correction) to examine differences in the observed level of optimism and pessimism between those with correct and incorrect answers on each question (*N* = 1244).

	Pessimism				Optimism			
	*M*_*True*_	*M*_*False*_	*t*	df	*M*_*True*_	*M*_*False*_	*t*	df
1. Effects of rinsing nose	19.97	19.70	0.68	1152.03	22.75	23.12	−1.52	1172.06
2. Efficacy of antibiotics in preventing COVID-19	19.78	23.27	−2.53*	26.07	22.92	22.42	0.44	25.61
3. The most common symptoms of COVID-19	19.43	20.86	−3.28**	675.03	22.96	22.79	0.63	657.09
4. Handwashing to protect from COVID-19	20.38	15.93	−8.10**	195.98	22.86	23.24	1.16	208.23
5. COVID-19 ways of transmission	19.21	20.56	−3.45**	1230.09	22.99	22.82	0.70	1196.67
6. The percentage of COVID patients that develop serious breathing problem	20.97	19.75	−1.72	126.16	22.51	22.95	0.88	120.81
7. Persons without symptoms can transmit COVID	19.76	22.80	−2.15**	40.56	22.94	21.85	1.18	40.41
8. Virus time of survival on surfaces	19.85	19.99	−0.17	85.12	22.87	23.46	−1.32	89.04

*p<0.05, **p<0.01.

Table 5 shows the correlation matrix between all observed variables. Age and education level were negatively correlated with pessimism and positively with optimism, whereas experiencing significant life event was positively correlated with anxiety, depression, and pessimism and negatively with optimism. Anxiety was positively correlated with pessimism and negatively with optimism. Only questions 4 and 5 satisfied the criteria for conducting the path analysis and testing mediation. Not all necessary associations between variables were significant on the remaining six questions. Therefore, the proposed mediation models were only tested for questions 4 and 5. A model proposing pessimism as a mediator in the relationship between anxiety and depression on the one side and the correct/incorrect answer on the questions on the other side (indirect path) was tested. Therefore, direct paths were included between anxiety/depression and pessimism and pessimism and correct/incorrect answer on question 4/question 5. An alternative direct path was added between depression/anxiety and the correct/incorrect answer. Optimism was not included in the models due to its non-significant relations with response accuracy.

**TABLE 5:** Correlations between the observed variables.

	Sex	Age	EC	LE	IPCM	CD	q1	q2	q3	q4	q5	q6	q7	q8	A	D	P
Sex	/																
Age	0.03	/															
Education	0.01	0.13**	/														
Significant life events	0.05	0.1.11**	0.03	/													
Prevention and control measures	−0.05	−0.05	−0.07*	0.09**	/												
Chronic diseases	−0.02	0.20**	−0.03	0.03	−0.03	/											
1. Effects of rinsing nose	−0.11**	−0.10**	0.07*	0.06*	0.04	0.00	/										
2. Efficacy of antibiotics in preventing COVID-19	0.04	−0.00	0.05	0.02	−0.00	−0.03	0.01	/									
3. The most common symptoms of COVID-19	0.06	−0.04	−0.00	−0.01	−0.01	−0.02	−0.09**	0.05	/								
4. Handwashing to protect from COVID-19	−0.04	−0.04	0.10**	0.07*	0.05	0.07*	0.16**	−0.05	−0.11**	/							
5. COVID-19 ways of transmission	0.05	−0.09**	−0.06*	−0.06*	−0.03	−0.08**	−0.06*	0.02	0.12**	−0.16**	/						
6. Breathing problem	−0.02	−0.03	0.05	0.02	0.00	0.08**	−0.03	0.01	−0.02	0.09**	−0.08**	/					
7. Persons without symptoms can transmit COVID	0.04	0.03	0.05	0.02	0.01	−0.01	−0.03	0.11**	0.06*	−0.02	−0.05	−0.01	/				
8. Virus time of survival on surfaces	0.07*	0.11**	0.03	0.00	−0.08**	0.03	−0.02	0.01	0.04	−0.04	−0.06*	0.01	0.13**	/			
Anxiety	0.15**	−0.03	−0.00	0.14**	0.03	0.15**	0.00	0.00	−0.04	0.17**	−0.10**	0.05	0.01	0.01	/		
Depression	0.08**	0.04	−0.04	0.11**	0.02	0.11**	−0.02	0.03	−0.02	0.10**	−0.04	0.07*	0.02	0.04	0.73**	/	
Pessimism	0.02	−0.06*	−0.06*	0.06*	0.03	0.09**	0.02	−0.08**	−0.09**	0.21**	−0.10**	0.05	−0.06*	−0.01	0.37**	0.33**	/
Optimism	0.01	0.07*	0.06*	−0.07**	−0.02	−0.04	−0.04	0.02	0.02	−0.03	0.02	−0.02	0.05	−0.03	−0.33**	−0.37**	−0.48**

Legend: * p<0.05, **p<0.01. EC, Education categorized; LE, Significant life events; IPCM, Infection prevention and control measures; CD, Chronic diseases; q1-q8, Questions on COVID-19 knowledge test; A, Anxiety; D, Depression; P, Pessimism; O, Optimism.

**FIGURE 1:**
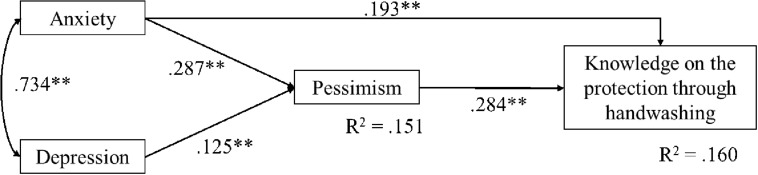
Display of the selected model of the relationship between mental health (anxiety and depression), pessimism and knowledge on the fourth question about handwashing as a mean of protection from COVID-19.

**FIGURE 2:**
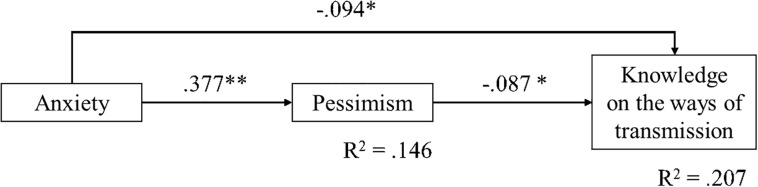
Display of the selected model of the relationship between mental health (anxiety), pessimism and knowledge on the fifth question on the ways of transmission.

Figures 1, 2 are displays of the estimated models shown to have all significant path coefficients, along with the lower WRMR index. Considering question 4, a full mediation model was tested (WRMR = 1.05) against an alternative partial mediation model, that is, adding direct paths between depression/anxiety and the correct/incorrect answer. There was no convergence to estimate such a partial mediation model, which was likely due to the higher than 0.70 correlation between anxiety and depression. Two other models were estimated, one adding only a direct path from depression to the accuracy of the answer (WRMR = 0.57) and the other adding only a direct path between anxiety and the answer (WRMR = 0.22). The last model was accepted and is shown in Figure 1. For question 5, depression was not included in the models due to a non-significant bivariate correlation with the category of the answer given (correct/incorrect). The WRMR of the full mediation model was 1.003, while the partial mediation model was a just identified model (WRMR = 0). Since the path coefficient between anxiety and the correct/incorrect answer on this question was significant, this model was selected (Figure 2). The indirect effects were estimated using the bootstrap method (maximum number of iterations = 1000; level of significance *p* < 0.05 and 95% confidence interval) and the obtained parameters are shown in Table 6. The estimated indirect effects of both models were significant.

**TABLE 6:** Standardized estimates and levels of significance of the estimated indirect path coefficients of the observed models, and the confidence intervals obtained with the bootstrap method.

Indirect paths	Estimate	Standard error	Estimate/standard error	*p*	95% Confidence interval
Anxiety→pessimism→ handwashing to protect from COVID-19 (Figure 1)	0.08**	0.02	4.38	0.000	0.05; 0.11
Depression→pessimism→ handwashing to protect from COVID-19 (Figure 1)	0.04**	0.01	2.90	0.004	0.02; 0.06
Anxiety→pessimism→ COVID-19 ways of transmission (Figure 2)	−0.03*	0.02	−2.23	0.026	−0.06; −0.01

**p < 0.01; *p < 0.05.

The selected model for question 4 (Figure 1) explained 16% of the variance of knowledge (the accuracy of the response to the question) and 15.1% of the variance of anxiety. Higher levels of knowledge were predicted with higher levels of anxiety and pessimism. A higher level of knowledge was also indirectly predicted by depression through a greater level of pessimism. The selected model for question 5 (Figure 2) explained 20.7% of the variance of knowledge and 14.6% of the variance of pessimism. Higher levels of knowledge were predicted by lower levels of anxiety and lower levels of pessimism and indirectly by anxiety through its relation with a higher level of pessimism.

## Discussion

This study aimed to examine the relationship between COVID-19 related knowledge and mental health (in terms of anxiety and depression) in a Croatian sample of participants. The results only partially confirmed our hypotheses. As expected, anxiety was positively correlated with pessimism and negatively with optimism, but optimism was not significantly associated with knowledge about COVID-19. Participants who were informed on COVID-19 symptoms, prevention through antibiotics (individuals who answered correctly on this question knew that the virus could not be prevented through antibiotics), and the mode of transmission were less pessimistic than uninformed participants. However, two questions—questions regarding handwashing as a protection from COVID-19 and serious breathing complications—yielded different results than the other questions. Namely, participants who responded correctly about handwashing had higher levels of anxiety, depression, and pessimism than those who responded incorrectly, whereas participants who responded correctly about the percentage of patients who develop serious respiratory problems had higher levels of depression than participants who did not know the answer to this question. Differences were not found in the rest of the questions nor at any question for the levels of optimism between participants who responded correctly and those whose answers were incorrect.

According to WHO, regularly practicing hand hygiene is the best way to be protected from the COVID-19 infection, and this information has been transmitted in mass media as well as by scientists (West et al., 2020). West et al. (2020) propose that the knowledge of effective hand hygiene provides individuals a proper level of capability, but this does not imply that people will have the opportunity to practice hand hygiene (e.g., have soap or hand sanitizer) or be motivated to do it (believe that this action is needed). Higher levels of anxiety, depression, and pessimism of participants who correctly answered the question on hand hygiene may be in line with this hypothesis. According to the participants’ responses to this question, it could be claimed that the vast majority of the sample possesses an appropriate level of knowledge on this behavior. However, according to the models presented here, being anxious was associated with higher levels of knowledge, and this might be due to anxiety referring to future events (e.g., people might ask themselves: Will I have the opportunity to wash my hands when needed? and Will others be motivated to wash hands when required?). Higher levels of depression of those who responded correctly may root from the fear of previous hand hygiene practices (e.g., Did I wash my hands when I was supposed to? or Did my children have enough soap at their school when it was still opened?). These findings should be compared with the findings of other researchers who studied the practicing of hand hygiene during SARS (Leung, 2003) and the current pandemic (Harper et al., 2020; Wang et al., 2020). Leung (2003) reported that participants with moderate but not mild and high levels of anxiety regularly do hand hygiene. Wang et al. (2020) found that hand hygiene contributes to lower levels of depression, anxiety, and stress in a Chinese sample. Both findings are not in line with findings of the study conducted on the Croatian sample, but Roy et al. (2020) consider frequently washing hands as a sign of anxiety. Moreover, in a study by Harper et al. (2020), fear of COVID-19 was the only predictor of positive behavior change (e.g., improved hand hygiene). Being aware that each individual is responsible for their own acts and behaviors (e.g., properly washing hands) might have resulted in higher levels of anxiety, depression, and pessimism in the participants in our study. Finally, it could be hypothesized that higher levels of pessimism may be related to knowing that handwashing can protect you and others and also knowing that it is not a habitual practice and that the population is not aware or motivated to do it.

However, the model presented for question 5 (ways of transmission) differs from previous findings, since participants with lower levels of anxiety are less pessimistic and better informed. According to Leung et al. (2004), there is a positive association between knowledge on the transmission of SARS and adopting precautionary measures, but Lau et al. (2007) found that misconceptions about the mode of transmission of the avian flu were associated with avoidance of hospitals, while Brug et al. (2004) found no association between behavior and SARS knowledge. It could only be hypothesized that, if a higher level of anxiety allowed the study participants to be more knowledgeable on preventive behaviors such as handwashing, at the same time it prevented them from acquiring precise information about the mode of transmission.

Considering the finding of higher levels of depression in participants who correctly answered the sixth question (percentage of patients developing serious respiratory problems), which also was the most difficult question in this study according to difficulty indexes, the centrality of accurate information on COVID-19 comes to the fore (Brooks et al., 2020). All around the world people are being given loads of information, and many of these pieces of information appeared to be misinformation (Huaxia, 2020). Public health experts in Croatia had been warning citizens that the geometrical growth of infected individuals would certainly lead to huge problems in hospitals, since a sufficient number of beds in intensive care units, as well as respirators, would have already been taken by patients with complications. This kind of information, although true and accurate, may contribute to increased depression (Rubin and Wessely, 2020; Wang et al., 2020) and, in addition, people with higher levels of depression may give particular attention to this kind of information and become obsessed by the search of the most catastrophic news that could confirm their worst expectations (Gao et al., 2020). Bearing this in mind, public health professionals have a huge responsibility when addressing citizens. Expert messages may sometimes lead to frightened citizens who may already be well informed of the worst consequences of the infection, because close people (family and friends) had suffered it. This may lead to broader knowledge, but at the same time it feeds the fear that it might happen to you or your loved ones.

Findings regarding pessimism in this study could be compared to the findings of Chang and Sivam (2004) who studied the relationship between direct pessimism and preventive health-related behaviors during the SARS epidemic in Singapore. Although the output variables in the proposed models differ, since our study measured COVID-19 related knowledge rather than practicing preventive behaviors, a few similarities occurred. In Chang and Sivam’s study (2004), participants with higher levels of defensive pessimism were experiencing higher levels of SARS-related fear and eventually practiced direct preventive behaviors. In the Croatian sample, higher levels of depression and anxiety are associated with higher levels of pessimism, and participants with higher levels of pessimism are better informed about the importance of handwashing.

An important finding of the study is that the directions of the observed relationships are different in the two proposed models. This finding might have a practical implication as it suggests that different types of information should be given regarding different knowledge on COVID-19. Specifically, if more information of the mode of transmission may be useful to reduce anxiety, this does not apply to some preventive behaviors such as handwashing. Thereby, in this latter case, it might be more useful to promote a behavioral change through persuasion, training, modeling, and enablement (West et al., 2020).

The study presented here has some limitations. The sample in this study is not a representative sample of the Croatian population. Only 14.5% out of 1244 participants were men, and lower educated persons are under-represented. Moreover, the study is cross-sectional, which does not allow examining how (and if) the mental health of the Croatian population changed during the pandemic, nor does it allow making conclusions of causality in the examined relationships among the variables. Moreover, anxiety and depression were not confirmed by a clinical psychologist. Future studies should include behavioral measures and try to collect data longitudinally.

Nevertheless, this study offers the first data on mental health during the COVID-19 pandemic in Croatia and proposes new models relating anxiety and depression with knowledge, also considering pessimism as a mediator. This is a promising research line for the implementation of health promotion strategies and clinical interventions by suggesting that knowledge about COVID-19 may be useful to reduce anxiety and depression, but must be differentiated according to the type of knowledge being promoted. Knowledge on the virus must be accurate and awareness must be promoted to reduce anxiety. However, too detailed information and an excessive focus on the catastrophic consequences of the infection and on the difficulty to receive appropriate and effective care may feed depression and pessimism. Finally, the promotion of health behaviors to reduce the risk of contagion may mainly be effective through behavioral change.

## Data Availability Statement

The datasets presented in this study can be found in online repositories. The names of the repository/repositories and accession number(s) can be found below: https://data.mendeley.com/datasets/4c7x83hd64/1?fbclid=IwAR2UtsRKQumAznipIIukVG1LMIkP80airnKiKz-n8gzjWBHTglg0r0RXviEMendeley, doi: 10.17632/4c7x83hd64.1.

## Ethics Statement

The studies involving human participants were reviewed and approved by the Ethics Committee of Department of Psychology, University of Zadar. The patients/participants provided their written informed consent to participate in this study.

## Author Contributions

MG, LM, and LS designed the study and collected the data. AŠ analyzed the data. SC contributed to the study design and supervised the whole process. All authors contributed to the article and approved the submitted version.

## Conflict of Interest

The authors declare that the research was conducted in the absence of any commercial or financial relationships that could be construed as a potential conflict of interest.

## Footnotes

1 https://www.who.int/emergencies/diseases/novel-coronavirus-2019/question-and-answers-hub/q-a-detail/q-a-coronaviruses
