# Supplementary material for: COVID-19 Related Knowledge and Mental Health: Case of Croatia
Source: Front Psychol. 2020 Nov 23;11:567368. doi: 10.3389/fpsyg.2020.567368 (PMC7726852; doi:10.3389/fpsyg.2020.567368)
Supplement: Supplementary file 1 [file Data_Sheet_1.docx]

**Supplementary Material**

**Demographic data**

1. Sex:

a) Male
b) Female

2. Age:

3. Highest completed level of education:
a) Elementary School
b) High School
c) Undergraduate level of study
d) Graduate level of study
e) Postgraduate level of study

4. Study orientation (if you are currently studying):

5. Have you experienced any significant life events in the last week (check the ones that apply to you):

a) Death of a close person

b) Termination of partnership

c) Changes in the work environment

d) Change of residence

e) I have not experienced a significant life event in the last week.

6. Are you under the care of an epidemiologist?

a) Yes

b) No

7. If under the measures of an epidemiologist, indicate what applies to you:

a) Self-isolation

b) Quarantine

8. Do you suffer from chronic diseases?

a) Yes

b) No

9. If you suffer from chronic diseases, indicate which one:

a) Diabetes
b) Heart disease
c) Respiratory disease

**COVID-19 knowledge test**

1. Rinsing nose regularly with saline solution:

a) has scientifically been proven to protect from COVID-19
b) prevents respiratory infections
c) both answers a) and b)
d) none of the above

2. Antibiotics are effective in preventing COVID-19. T/F

3. The most common symptoms of COVID-19 are:

a) fever
b) tiredness
c) dry cough
d) all of the above (a, b & c)
e) none of the above

4. The best way to protect from COVID-19 is to wash hands regularly. T/F

5. COVID-19 is transmitted by:

a) mosquito bite
b) pets
c) air
d) none of the above

*** At the time of conducting research there was no evidence that pets transmit COVID-19

6. What is the percentage of COVID patients that develop serious breathing problems?

a) around 17%
b) around 8%
c) around 22%
d) none of the above

7. Persons without symptoms can transmit COVID. T/F

8. On surfaces, the virus can survive:

a) few hours
b) few weeks
c) the virus can not survive on surfaces
